# Apply Woods Model in the Predictions of Ambient Air Particles and Metallic Elements (Mn, Fe, Zn, Cr, and Cu) at Industrial, Suburban/Coastal, and Residential Sampling Sites

**DOI:** 10.1100/2012/207620

**Published:** 2012-04-24

**Authors:** Guor-Cheng Fang, Ci-Song Huang

**Affiliations:** Department of Safety, Health and Environmental Engineering, Hung Kuang University, Sha-Lu, Taichung 433, Taiwan

## Abstract

The main purpose for this study was to monitor ambient air particles and metallic elements (Mn, Fe, Zn, Cr, and Cu) in total suspended particulates (TSPs) concentration, dry deposition at three characteristic sampling sites of central Taiwan. Additionally, the calculated/measured dry deposition flux ratios of ambient air particles and metallic elements were calculated with Woods models at these three characteristic sampling sites during years of 2009-2010. As for ambient air particles, the results indicated that the Woods model generated the most accurate dry deposition prediction results when particle size was 18 **μ**m in this study. The results also indicated that the Woods model exhibited better dry deposition prediction performance when the particle size was greater than 10 **μ**m for the ambient air metallic elements in this study. Finally, as for Quan-xing sampling site, the main sources were many industrial factories under process around these regions and were severely polluted areas. In addition, the highest average dry deposition for Mn, Fe, Zn, and Cu species occurred at Bei-shi sampling site, and the main sources were the nearby science park, fossil fuel combustion, and Taichung thermal power plant (TTPP). Additionally, as for He-mei sampling site, the main sources were subjected to traffic mobile emissions.

## 1. Introduction

Dry deposition is the process by which an atmospheric air pollutant is transferred to the surface of the earth as a result of the turbulent motion of air [1, 2]. The concentrations and size distributions of trace metals are governed by the nature of emissions into the atmosphere as well as by the rates of wet and dry deposition, cloud processing, chemical transformations, and the exchange of air between the boundary layer and free troposphere [3]. It occurs as trace gases and particles are adsorbed or react on objects (plants, soil, water, buildings, etc.) at the earth's surface. Factors that govern dry deposition of gases and particles include atmospheric turbulence intensity, the nature of the gas and particles, and that of the surface [4].

In attrition, fossil fuel and wood combustion, as well as waste incineration and industrial processes, are the main anthropic sources of metals to the atmosphere [5]. Natural emissions (from crustal minerals, forest fires, and oceans) and industrial processes (fuel combustion, waste incineration, automobile exhaust, and mining, and quarrying) are the principal sources of the metals in ambient air. Traffic emissions also represent various potential sources of metals, including combustion products from fuel and oil, road construction materials, road dust, and wear products from tires, brake linings, and bearings [6].

Furthermore, air pollution has become a more and more serious problem in Taiwan, especially in central Taiwan. It includes aerosol, effluvium, secondary pollutant, and organic solvent vapor [7]. In Taiwan, the number of vehicles on the main roads has increased in recent decades. Motor vehicle exhaust is an important source of fine particles [8]. Heavy metals such as Pb, Cu, and Zn mainly are found in the particulate phase [9].

Moreover, considerable research has been conducted to investigate the dry deposition of air pollutants. Among these, heavy metals are of particular interest as most of them are toxic to humans and ecosystems. Dry deposition may be particularly important near urban/industrial areas adjacent to surface waters where particle concentrations and pollutants associated with them are relatively high [10].

Therefore, atmospheric particulates and associated trace metals have been linked with both short- and long-term adverse health effects which mostly include chronic respiratory diseases, heart diseases, lung cancer, and damage to other organs [11–14]. The combined model resolved 97% of PM_10_ mass concentrations and the evaluation analysis showed the results obtained by the combined model were reasonable [15]. The concept of the dry deposition velocity of an air pollutant has been widely utilized/used in modeling air pollution [16, 17].

The goal of this study was (1) monitoring and modeling ambient air particulates and metallic elements Mn, Fe, Zn, Cr, and Cu concentration, dry deposition at three characteristic (suburban/coastal, residential, and industrial) sampling sites; (2) finding the optimum particle size that performs better results with respect to Woods model in the prediction of dry depositions for ambient air particles and metallic elements (Mn, Fe, Zn, Cr, and Cu) in central Taiwan.

## 2. Dry Deposition Model

Atmospheric particles with aerodynamic diameters <10 *μ*m (PM_10_) have been under scrutiny as they are easily inhaled and deposited within the respiratory system [18]. Studies show that PM_10_ has a role in the incidence and severity of respiratory diseases [19, 20]. Therefore, 10 *μ*m was used to calculate *V*_*d*_ (Dry deposition Velocity) and modeled dry deposition for comparison with measured dry deposition fluxes at the three characteristic sampling sites.

The descriptions of one model are all described as follows.

### 2.1. Empirical Equations: Deposition Model by Woods [21, 22]

The deposition across the entire range of particle sizes may be predicted by simple empirical equations and be applied to vertical surfaces across all deposition regimes when configured in the following manner [23]:

(1) $$\begin{array}{c} V_{d}^{+}=k_{1}{Sc}^{-2/3}+k_{2}\pi^{+2\;}\;\text{if}\;k_{1}{Sc}^{-2/3}+k_{2}\tau^{+2}\leq k_{3},\\ V_{d}^{+}=k_{3}\;\text{if}\;k_{1}{Sc}^{-2/3}+k_{2}\tau^{+2}>k_{3}. \end{array}$$

Table 1 summarizes different values for *k*_1_, *k*_2_, and *k*_3_ found by different investigators [24].

To apply empirical equations of this type to horizontal surfaces, a simple modification to accounts for the effect of gravitational settling on the particle deposition velocity [25]:

(2) $$V_{d}^{+}=k_{1}{Sc}^{-2/3}+k_{2}\tau^{+2}+g^{+}\tau^{+},$$

where *g* ^+^ is the dimensionless gravitational acceleration defined by

(3) $$g^{+}=\frac{gv}{u_{\tau }^{3}},$$

and *g* is positive for a floor and negative for a ceiling surface. In (2) the first term on the right hand side accounts for Brownian diffusion, the second term accounts for interactions between particle inertia and turbulent eddies and the final term accounts for gravitational settling. However, (2) does not account for surface roughness. In the following (2) is referred to as Woods model.

The previously mentioned equation has been described in detail in previous studies [24].

Average particle sizes of 1.0, 2.5, 10, and 18 *μ*m in TSP were selected in this study to model the particle-bound dry deposition fluxes. Then, the calculated dry deposition velocities are multiplied by the measured ambient air concentrations to obtain calculated dry deposition fluxes for ambient air particles and metallic elements (Mn, Fe, Zn, Cr, and Cu). The calculated dry deposition fluxes are then compared with the measured dry deposition fluxes. The calculated/measured flux ratios for dry deposition were then used to determine whether the over—or under—estimates dry deposition fluxes.

## 3. Method

### 3.1. Sampling Program

**Figure 1:**
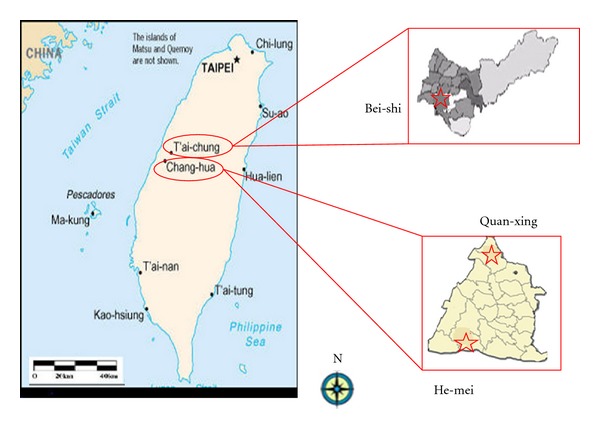
Geographical location for three characteristic sampling sites in central Taiwan.

Figure 1 lists the five characteristic sampling sites. All samples were obtained in 1350–1400 min during the one-day sampling period for each sampling group. They are designated as follows.

The Bei-shi (suburban/coastal) sampling station (24°13′31.82′′N, 120°34′09.45′′E), which is in Sha-lu, Taichung, Taiwan, is a suburban/coastal station with no nearby obstructions. The immediate vicinity is residential, with an expressway with heavy traffic located approximately 2 km east of the station.

The He-mei (residential) town sampling station (24°06′00.54′′N, 120°30′51.34′′E) is located in a residential area. The main pollution sources are resident activities and vehicular emissions. The Quan-xing (industrial) sampling station (24°08′37.89′′N, 120°29′09.43′′E) is located in Shen-kang, a town covering 246.8 hectares, roughly half of which, 126.5 hectares, is occupied by factories and industry. Additionally, the Taichung Thermal Power Plant (TTPP) sits on 281 hectares of the wetland along the coast, on the western side of the sampling site. This plant burns coal to supply central Taiwan with 4,400 MW of electricity daily.

### 3.2. Sampling Program

#### 3.2.1. PS-1 (Total Suspended Particulate) Sampler

The PS-1 is a complete air sampling system designed to collect suspended airborne particles (GMW High-Volume Air Sampler; Graseby-Andersen, USA). Maximum particle size in this study was roughly 100 *μ*m. Sampler flow rate was 200 L/min. A quartz filter 10.2 cm in diameter filtered suspended particles. Filters were first conditioned for 24 h under an electric chamber under a humidity 35 ± 5% and temperature 25 ± 5°C prior to both on and off weighing. Filters were in a sealed CD box when transported and stored. The sampling device and procedures are similar to those in previous study [26].

#### 3.2.2. Dry Deposition Plate

The dry deposition plate (DDP) had a smooth horizontal surrogate surface, providing a lower-bound estimate of dry deposition flux. The polyvinyl chloride (PVC) DDP measured 21.5 cm long, 8.0 cm wide by 0.8 cm thick. The DDP also had a sharp leading edge that was pointed into the prevailing wind. All filters were maintained under 50% relative humidity at 25°C for over 48 h. Prior to sampling, all filters were weighed to 0.0001 Gram-significant digits [26].

### 3.3. Chemical Analysis

Samples were placed in an oven for 12 h before being weighed. One quarter of each filter was cut and selected before the digestion process. The filters were then cut into thin pieces and placed in a Teflon cup. Then, 3 mL hydrochloric acid (HCl) and 9 mL nitrate (HNO_3_) were mixed together and then poured into this cup. Samples were then heated at 50°C on a hotplate for 2 h. Samples after digestion on the hotplate were then filtered. Following filtration, the sample solution was added to 0.2% HNO_3_ to create 100 mL solution. The samples were maintained at 4°C in the refrigerator for ICP-AES analysis. Concentrations of metallic elements Mn, Fe, Zn, Cr, and Cu were measured in this study. ICP-AES was conducted using a Perkin Elmer Optima 2100 Plasma Emission Spectrometer to analyze the metallic elements. A time and an argon gas plasma flow rate of 15 L/min were applied. The nebulizer flow rate was set to 0.65 L/min and the sample flow rate was set to 1.5 mL/min.

### 3.4. Quality Control

Blank test background contamination was assessed using operational blanks (unexposed projection film and a quartz filter) that were processed simultaneously with field samples. The field blanks were exposed in the field when the field sampling box was opened to remove and replace filters. This study accounted for background contamination of metallic elements by subtracting field blank values from concentrations. Field blank values were extremely low, generally below or around the method detection limits. In this study, the background contamination is insignificant and can be ignored. Blank test results were 0.21, 0.35, 0.19, 0.22, and 0.25 *μ*g for Mn, Fe, Zn, Cr, and Cu. Respectively.

## 4. Results and Discussion

The meteorological parameters were measured by using weather station Model 525 (Spectrum Technologies, Inc., Taichung County, Taiwan).

Table 1 shows the meteorological conditions and average metallic elements (Mn, Fe, Zn, Cr, and Cu) in total suspended particulates (TSPs) and dry deposition at three characteristic sampling sites during 2009-2010.

 The results indicated that the average temperature, relative humidity, and wind speed were 24.1°C, 76.9%, and 1.9 m/sec at Bei-shi sampling site, respectively. The results also showed that the average temperature, relative humidity, and wind speed were 24.5°C, 75.2%, and 2.2 m/sec at He-mei sampling site, respectively. Finally, as for Quan-xing sampling site, the results indicated that the average temperature, relative humidity, and wind speed were 24.4°C, 78.3%, and 2.6 m/sec, respectively.

As for metallic element Mn, the results indicated that the average concentrations order in TSP for location variations was Quan-xing (industrial) > He-mei (residential) > Bei-shi (suburban/coastal) and the average dry deposition order was Bei-shi (suburban/coastal) > Quan-xing (industrial) > He-mei (residential) (Table 2). Furthermore, for metallic element Fe, the results also indicated that the average concentration order in TSP for location variations was Quan-xing (industrial) > Bei-shi (suburban/coastal) > He-mei (residential) and the average dry deposition order was Bei-shi (suburban/coastal) > Quan-xing (industrial) > He-mei (residential). Moreover, metallic element Zn, the results indicated that the average concentration order in TSP for location variations was Quan-xing (industrial) > Bei-shi (suburban/coastal) > He-mei (residential) and the average dry deposition order was Bei-shi (suburban/coastal) > Quan-xing (industrial) > He-mei (residential). Besides, for metallic element Cr, the results also indicated that the average concentration order in TSP for location variations was He-mei (residential) > Quan-xing (industrial) > Bei-shi (suburban/coastal) and the average dry deposition order was He-mei (residential) > Bei-shi (suburban/coastal) > Quan-xing (industrial). Finally, metallic element Cu, the results indicated that the average concentration order in TSP for location variations was Quan-xing (industrial) > Bei-shi (suburban/coastal) > He-mei (residential) and the average dry deposition order was Bei-shi (suburban/coastal) > He-mei (residential) > Quan-xing (industrial).

**Figure 2:**
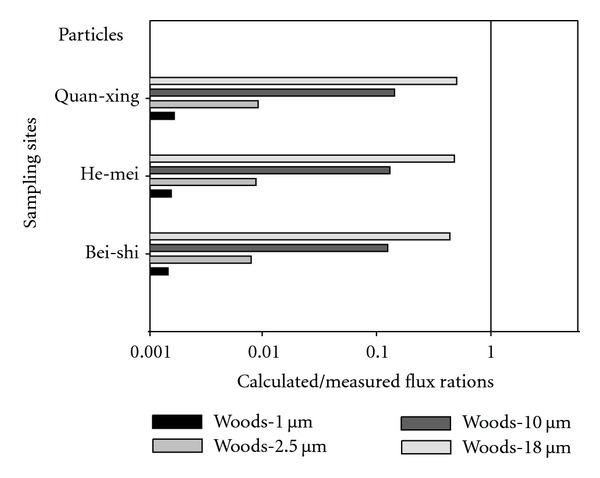
It displays the average calculated/modeled ratios results for ambient air particles by using Woods models for various particles sizes (1.0 *μ*m, 2.5 *μ*m, 10 *μ*m, and 18 *μ*m) at three sampling sites.

Figure 2 displayed the average calculated/modeled ratios results for ambient air particles by using Woods models for various particles sizes (1.0 *μ*m, 2.5 *μ*m 10 *μ*m, and 18 *μ*m) at three sampling sites. The results indicated that the average calculated/measured flux ratios for particle sizes by using Woods model for (1.0 *μ*m, 2.5 *μ*m 10 *μ*m, and 18 *μ*m) particle size were 0.0014, 0.0079, 0.1210, and 0.4381 at Bei-shi sample site, respectively. And the average calculated/measured flux ratios for particle sizes by using Woods model for (1.0 *μ*m, 2.5 *μ*m, 10 *μ*m, and 18 *μ*m) particle size were 0.0016, 0.0089, 0.1330, and 0.4792 at He-mei sample site, respectively. Moreover, the results indicated that the average calculated/measured flux ratios for particle sizes by using Woods model for (1.0 *μ*m, 2.5 *μ*m, 10 *μ*m, and 18 *μ*m) particle size were 0.0017, 0.0093, 0.1411, and 0.5027 at Quan-xing sample site, respectively.

**Figure 3:**
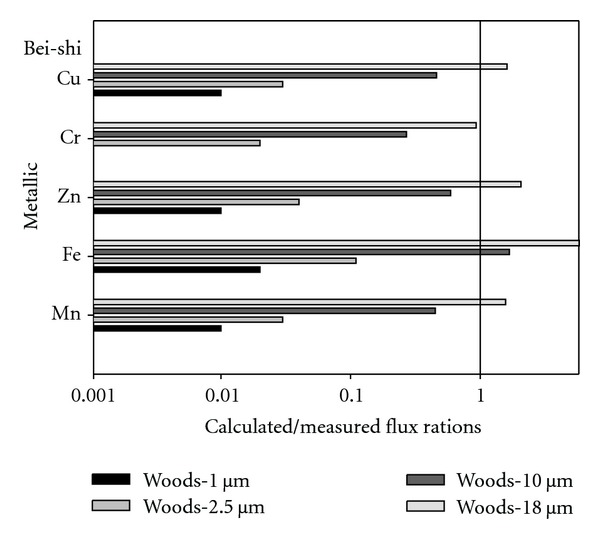
It displays the average calculated/measured flux ratios by using Woods models in the prediction of ambient air metallic elements dry deposition for various particle sizes (1 *μ*m, 2.5 *μ*m, 10 *μ*m, and 18 *μ*m) at Bei-shi sample site.

Figure 3 displayed the average calculated/measured flux ratios by using Woods models in the prediction of ambient air metallic elements dry deposition for various particle sizes (1 *μ*m, 2.5 *μ*m, 10 *μ*m, and 18 *μ*m) at Bei-shi sample site. The results indicated that the average calculated/measured flux ratios for metallic elements Mn, Fe, Zn, Cr, and Cu by using Woods model for 1 *μ*m particle size were 0.01, 0.02, 0.01, 0, and 0.01, respectively. And the average calculated/measured flux ratios for metallic elements Fe, Zn, Cr, and Cu by using Woods model for 2.5 *μ*m particle size were 0.03, 0.11, 0.04, 0.02, and 0.03, respectively. In addition, the results indicated that the average calculated/measured flux ratios for metallic elements Mn, Fe, Zn, Cr, and Cu by using Woods model for 10 *μ*m particle sizes were 0.45, 1.68, 0.59, 0.27, and 0.46, respectively. Finally, the results also indicated that the average calculated/measured flux ratios for metallic elements Mn, Fe, Zn, Cr, and Cu by using Woods model for 18 *μ*m particle size were 1.57, 5.87, 2.06, 0.93, and 1.61 at Bei-shi sample site, respectively.

**Figure 4:**
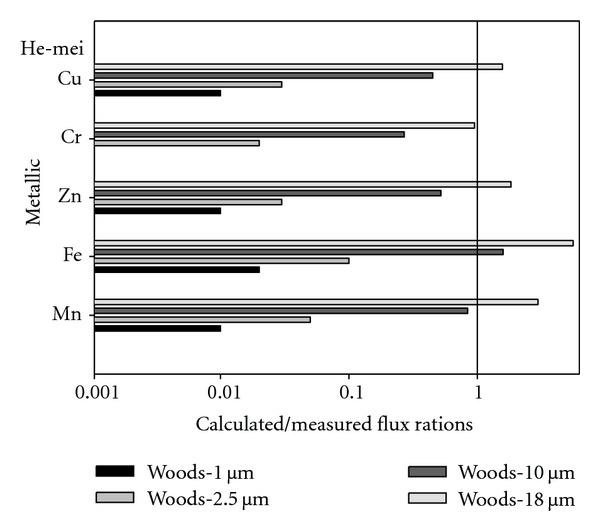
It displays the average calculated/measured flux ratios by using Woods models in the prediction of ambient air metallic elements dry deposition for various particle sizes (1 *μ*m, 2.5 *μ*m, 10 *μ*m, and 18 *μ*m) at He-mei sample site.

Figure 4 displayed the average calculated/measured flux ratios by using Woods models in the prediction of ambient air metallic elements dry deposition for various particle sizes (1 *μ*m, 2.5 *μ*m, 10 *μ*m, and 18 *μ*m) at He-mei sample site. The results indicated that the average calculated/measured flux ratios for metallic elements Mn, Fe, Zn, Cr, and Cu by using Woods model for 1 *μ*m particle size were 0.01, 0.02, 0.01, 0, and 0.01, respectively. And the average calculated/measured flux ratios for metallic elements Fe, Zn, Cr, and Cu by using Woods model for 2.5 *μ*m particle size were 0.05, 0.10, 0.03, 0.02, and 0.03, respectively. Furthermore, the results indicated that the average calculated/measured flux ratios for metallic elements Mn, Fe, Zn, Cr, and Cu by using Woods model for 10 *μ*m particle sizes were 0.84, 1.59, 0.52, 0.27, and 0.45, respectively. Finally, the results also indicated that the average calculated/measured flux ratios for metallic elements Mn, Fe, Zn, Cr, and Cu by using Woods model for 18 *μ*m particle size were 2.96, 5.57, 1.83, 0.95, and 1.57 at He-mei sample site, respectively.

**Figure 5:**
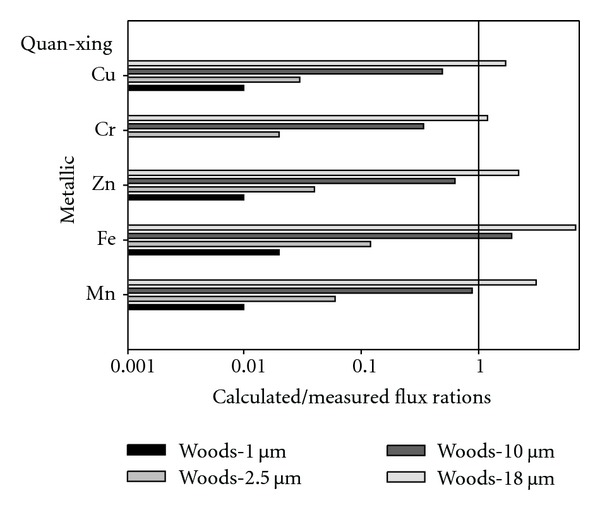
It displays the average calculated/measured flux ratios by using Woods models in the prediction of ambient air metallic elements dry deposition for various particle sizes (1 *μ*m, 2.5 *μ*m, 10 *μ*m, and 18 *μ*m) at Quan-xing sample site.

Figure 5 displayed the average calculated/measured flux ratios by using Woods models in the prediction of ambient air metallic elements dry deposition for various particle sizes (1 *μ*m, 2.5 *μ*m, 10 *μ*m, and 18 *μ*m) at Quan-xing sample site. The results indicated that the average calculated/measured flux ratios for metallic elements Mn, Fe, Zn, Cr, and Cu by using Woods model for 1 *μ*m particle size were 0.01, 0.02, 0.01, 0, and 0.01, respectively. And the average calculated/measured flux ratios for metallic elements Fe, Zn, Cr, and Cu by using Woods model for 2.5 *μ*m particle size were 0.06, 0.12, 0.04, 0.02, and 0.03, respectively. Moreover, the results indicated that the average calculated/measured flux ratios for metallic elements Mn, Fe, Zn, Cr, and Cu by using Woods model for 10 *μ*m particle sizes were 0.88, 1.91, 0.63, 0.34, and 0.49, respectively. Finally, the results also indicated that the average calculated/measured flux ratios for metallic elements Mn, Fe, Zn, Cr, and Cu by using Woods model for 18 *μ*m particle size were 3.09, 6.69, 2.19, 1.19, and 1.70 at Quan-xing sample site, respectively.

From the above analysis, the results indicate that the Woods model generated the most accurate dry deposition prediction results for ambient air particles when particle size was at 18 *μ*m in this study.

The results also indicated that the Woods model exhibited better dry deposition prediction performance for the ambient air metallic elements (i.e., Mn, Fe, Zn, Cr, and Cu) when the particle size was at 10 *μ*m in this study. As for the meteorological conditions, average temperature, humidity, wind direction, and wind speed did not display any significant variations. Finally, the summarized important issue is that the highest average concentrations of Mn, Fe, Zn, and Cu species in TSP occurred at Quan-xing (industrial) sampling site, with many industrial factories under process around these regions that were severely polluted areas. In addition, the summarized important issue is that the highest average dry deposition for Mn, Fe, Zn, and Cu species occurred at Bei-shi (suburban/coastal) sampling site, and the main sources were the nearby science park, fossil fuel combustion, and Taichung thermal power plant (TTPP). Additionally, the summarized important issue is that the highest average concentrations of Cr species in TSP and dry deposition occurred at He-mei (residential) sampling site, and the main sources were subjected to traffic mobile emissions.

## 5. Conclusions

The main conclusions for this study are listed as follows.

1. As for ambient air particles, the modeling results showed that the Woods model exhibits better average calculated/modeled ratios for 18 *μ*m particle size at all sampling sites of this study.
2. As for metallic elements Mn, Fe, Zn, and Cu, the results showed that the Woods model exhibits better dry deposition flux results for 10 *μ*m particle size at Bei-shi, Quan-xing, and He-mei sampling sites. However, as for metallic element Cr, the results showed that the Woods model exhibits better dry deposition flux results for 18 *μ*m particle size at all sampling sites.
3. As for the these three sampling sites, the results indicated that the average highest metallic elements Mn, Fe, Zn, Cr, and Cu concentrations in TSP and dry deposition were occurred at Quan-xing (industrial) and Bei-shi (suburban/coastal) areas, respectively, and that average lowest metallic elements (Mn, Fe, Zn, Cr, and Cu) concentrations in TSP and dry deposition occurred at Bei-shi (suburban/coastal) and Quan-xing (industrial) area, respectively.
4. From the point of view of metallic elements concentrations, metallic element Fe has the average highest metallic elements concentrations for any of sampling site in this study. And the average lowest metallic elements concentrations were Mn, Zn, and Cr at Bei-Shi, He-mei, and Quan-xing sampling sites, respectively. From the point of view of dry deposition, the average lowest dry deposition Cr at both Bei-shi and Quan-xing sampling sites while the lowest metallic element dry deposition at Her-Mei sampling site was Zn.

## Figures and Tables

**Table 1:** Recommended values of *k*_1_, *k*_2_ and *k*_3_ for (1), [24].

*k*_1_	*k*_2_	*k*_3_	Investigator
0.084			Cleaver and Yates
0.059			Friedlander
0.045	4.5 × 10^−4^	0.13	Wood

0.075		0.3	Davies
0.07	3.5 × 10^−4^	0.18	Papavergos and Hedley

	3.79 × 10^−4^		Kneen and Strauss
	6 × 10^−4^		Liu and Agarwal
		0.14	Fan and Ahmadi

**Table 2:** Meteorological conditions and average metallic elements (Mn, Fe, Zn, Cr, and Cu) in total suspended particulates (TSPs) and dry deposition at three characteristic sampling sites during year 2009-2010.

	Bei-shi (suburban/coastal) Average (*N* = 60)	He-mei (residential) Average (*N* = 60)	Quan-xing (industrial) Average (*N* = 60)
Temp (°C)	24.1	24.5	24.4
RH (%)	76.9	75.2	78.3
WS (m/s)	1.9	2.2	2.6
PWD	SE	SE	SE

	TSP (ng/m^3^)	TSP (ng/m^3^)	TSP (ng/m^3^)

Mn	16.0	60.1	80.8
Fe	290.5	93.7	2698.6
Zn	34.3	23.5	110.1
Cr	17.5	78.9	20.1
Cu	39.6	31.9	68.4

	Dry deposition (ng/m^2^/min)	Dry deposition (ng/m^2^/min)	Dry deposition (ng/m^2^/min)

Mn	36.3	14.1	17.6
Fe	2547.8	34.9	311.6
Zn	103.6	17.2	44.2
Cr	23.5	36.4	10.4
Cu	89.9	35.1	26.5

Temp: Temperature, RH: relative humidity, WS: wind speed, PWD: prevailing wind.
